# Comparison of the clinical characteristics in parents and their children in a series of family clustered *Mycoplasma pneumoniae* infections

**DOI:** 10.1186/s12890-024-02922-0

**Published:** 2024-03-04

**Authors:** Xu Liu, Qingfeng Zhang, Hao Chen, Yueying Hao, Jingyi Zhang, Shiqian Zha, Beini Zhou, Yaohua Yi, Rui Xiao, Ke Hu

**Affiliations:** 1 Department of Respiratory and Critical Care Medicine, Renmin Hospital of Wuhan University, Wuhan, 430060 China; 2 School of Remote Sensing and Information Engineering, Wuhan University, Wuhan, 430079 China; 3 Research Center of Digital Imaging and Intelligent Perception, Wuhan University, Wuhan, 430079 China

**Keywords:** *Mycoplasma pneumoniae*, Clustering, Adult, Children

## Abstract

**Background:**

*Mycoplasma pneumoniae* infections have increased in China recently, causing some evidence of familial clustering. The purpose of this study was to compare the clinical features of parents and children in cases of familial clustering of *Mycoplasma pneumoniae* infection.

**Methods:**

A retrospective analysis was performed on the cases of familial clustering of *Mycoplasma pneumoniae* infection, and the clinical characteristics of parents and children were compared.

**Results:**

We identified 63 families, of these, 57 (65.5%) adults and 65 (94.2%) children required hospitalization. Fifty-seven adults (mean age 35.1 ± 4.6 years, 80.7% female) and 55 children (mean age 6.3 ± 3.9 years, 54.5% female) were included in the analysis. The incidence of mycoplasma infection in adults had increased gradually over the past year, while the rate in children had spiked sharply since June 2023. The clinical symptoms were similar in the two groups, mainly fever and cough. The peak temperature of children was higher than that of adults (39.1 ± 0.7℃ vs 38.6 ± 0.7℃, *p* = 0.004). Elevated lactate dehydrogenase was more common in children than in adults (77.8% vs 11.3%, *p* < 0.001). Bronchial pneumonia and bilateral involvement were more common in children, while adults usually had unilateral involvement. Three (60%) adults and 21 (52.5%) children were macrolide-resistant *Mycoplasma pneumoniae* infected. Children were more likely to be co-infected (65.5% vs 22.8%, *p* < .001). Macrolides were used in most children and quinolones were used in most adults. Ten (18.2%) children were diagnosed with severe *Mycoplasma pneumoniae* pneumonia, whereas all adults had mild disease. Children had a significantly longer fever duration than adults ((5.6 ± 2.2) days vs (4.1 ± 2.2) days, *p* = 0.002). No patient required mechanical ventilation or died.

**Conclusions:**

*Mycoplasma pneumoniae* infection shows a familial clustering epidemic trend at the turn of summer and autumn, with different clinical characteristics between parents and children.

**Supplementary Information:**

The online version contains supplementary material available at 10.1186/s12890-024-02922-0.

## Introduction

Recently, there is an increased incidence of *Mycoplasma pneumoniae* pneumonia (MPP) in China, which causes sporadic cluster infections in communities, families or congregated settings [1]. *Mycoplasma pneumoniae* (MP) is the most common pathogen detected in community-acquired pneumonia in China [2]. MPP is a common respiratory infection worldwide. The incidence can increase several-fold during epidemic years, and outbreaks are often reported in schools, military camps, and other congregated settings [3]. Although older children and adolescents are mainly affected, infections and diseases caused by MP occur in all age groups [4]. Previous studies have reported that children and adults may have different manifestations of MPP, especially in terms of chest radiography findings [5, 6].

The aim of this study was to describe the characteristics of recent sporadic clusters of mycoplasma pneumonia and to compare the clinical features between adults and children.

## Materials and methods

### Study design

This study is a retrospective analysis that collected data on familial clusters of *Mycoplasma pneumoniae* pneumonia among inpatients from August to October 2023 at Renmin Hospital of Wuhan University in China. And we calculated the infection rates of MP in adults and children hospitalized in our hospital from October 2022 to October 2023 by accessing the electronic medical record system. We categorized individuals into adults and children within the familial clusters and conducted a comparative analysis of the clinical features between these two groups. The Ethics Committee of Renmin Hospital of Wuhan University approved the study. Since this was a retrospective study, the Ethics Committee of Renmin Hospital of Wuhan University, agreed to exempt subjects from the informed consent.

### Inclusion and exclusion criteria

Inclusion criteria: Patients who met the definition of familial cluster MP infection were included in the study. Familial cluster cases were defined as a situation where a minimum of two or more individuals from the same family were infected with MP, and the infection spanned across at least two generations. Infections among family members were classified as concurrent if they occurred within a 3-day period, and as sequential if they occurred within a 3 to 14-day period. The diagnostic criteria for *Mycoplasma Pneumoniae* Pneumonia (MPP) were as follows: Patients exhibited clinical symptoms indicative of a respiratory tract infection, such as fever, cough, and dyspnea. Radiological examinations revealed abnormalities in the chest. Additionally, the pathogenic test for MP yielded a positive result which was determined by any of the following: MP-IgM titers exceeding 1:160, a positive MP-DNA result, or the detection of MP through metagenomic next-generation sequencing. MP infection was defined as a positive MP result, in the absence of infiltrations on imaging. The diagnosis of adult severe *Mycoplasma pneumoniae* pneumonia (SMPP) was based on the 2016 Chinese guidelines for the diagnosis and treatment of adult community-acquired pneumonia [7]. Diagnosis of SMPP in children according to the Guidelines for the diagnosis and treatment of *Mycoplasma pneumoniae* pneumonia in children (2023 edition) [8].

Exclusion Criteria: Outpatients and inpatients who lacked access to complete information, such as records from hospitalizations at other institutions, were excluded from the study.

### Pathogenic detection methods

Assessing MP-specific IgM antibodies in plasma, with a titer of 1:160 or higher considered positive. Obtaining positive results from MP polymerase chain reaction (PCR) tests conducted on respiratory specimens. Detecting positive MP-DNA in bronchoalveolar lavage fluid using metagenomic next-generation sequencing. Macrolide-resistant *Mycoplasma pneumoniae* (MRMP) is detected by identifying specific single-nucleotide mutations in the V region of the 23S rRNA gene of the *M. pneumoniae* genome, which are indicative of macrolide resistance.

### Outcomes

The study outcomes encompassed demographic attributes, results from laboratory tests, findings from lung imaging studies, outcomes of pathogen-specific laboratory tests, the status of treatments administered, and the treatment outcomes observed in both cohorts.

### Data analysis

SPSS v26 software (IBM Statistics, Armonk, NY) was used for data analysis. Continuous data are expressed as mean ± standard deviation, with Student’s t-test. Categorical data are represented by the number of cases (%) for comparison of differences between groups using the non-parametric chi-squared test. A 2-sided alpha level of 0.05 was considered statistically significant.

## Results

### Epidemiology information and symptoms in both groups

**Fig. 1:**
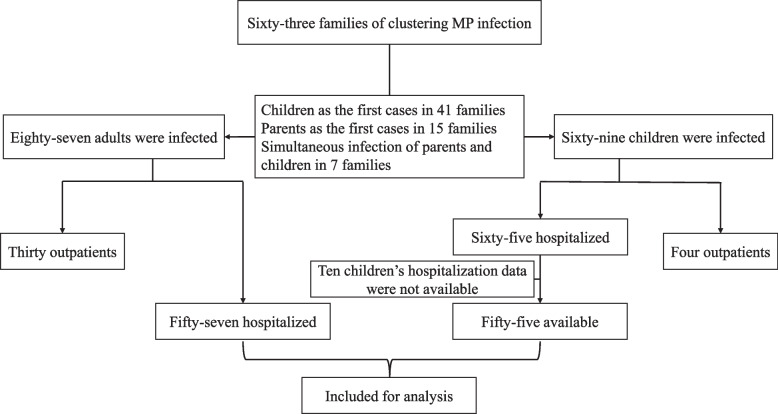
Research flowchart

We identified 63 family clusters of MP infection between August 2023 and October 2023, with one cluster involving three generations. These clusters comprised 87 adults and 69 children. In 41 (65.1%) families, the children were the first to be infected, followed by their parents who took care of them. In 15 (23.8%) families, the children were infected after the adults. Seven (11.1%) families had parents and children infected at the same time. Of these adults, 57 (65.5%) required hospitalization, 30 (34.5%) received outpatient or community care. Among the children, 65 (94.2%) were hospitalized (10 of them were admitted to another hospital and no data were available), and 4 (5.8%) were treated as outpatient. Finally, the parents group consisted of 57 patients (mean age 35.1 ± 4.6 years, 80.7% female) and the children group consisted of 55 patients (mean age 6.3 ± 3.9 years, 54.5% female) (Fig. 1).

**Fig. 2:**
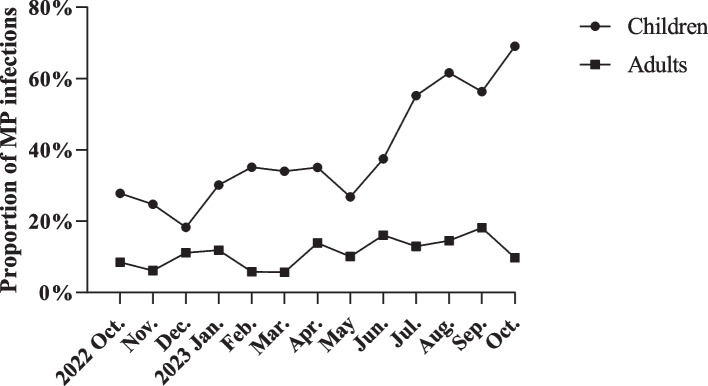
The epidemiological trends of MP infection in adults and children from October 2022 to October 2023

Then, we observed a sharp increase in the incidence of mycoplasma infection in children after June 2023, reaching a peak of 69.1% in October 2023. In contrast, the incidence of mycoplasma infection in adults showed a gradual increase from October 2022 to October 2023, with a maximum of 18.2% in September (Supplemental Table 1 and Fig. 2).

**Table 1:** Demographics and symptoms

Variables	Parents group^b^ (*n* = 57)	Children group^b^ (*n* = 55)	χ^2^/t value	*p* value
Age^a^, year	35.1 ± 4.6	6.3 ± 3.9	NA	NA
Female, n (%)	46 (80.7)	30 (54.5)	8.780	**0.003**
Fever, n (%)	42 (73.7)	53 (96.4)	11.183	**0.001**
Maximum temperature, ℃	38.6 ± 0.7	39.1 ± 0.7	2.961	**0.004**
Chill, n (%)	12 (21.1)	11 (20)	0.019	0.890
Cough, n (%)	57 (100)	55 (100)	NA	NA
Sputum production, n (%)	50 (87.7)	34 (61.8)	10.015	**0.002**
Shortness of breath, n (%)	12 (21.1)	7 (12.7)	1.377	0.241
Rhinorrhea, n (%)	6 (10.5)	9 (16.4)	0.822	0.365
Headache, n (%)	21 (36.8)	2 (3.6)	18.913	** < .001**
Sore throat, n (%)	12 (21.1)	2 (3.6)	7.763	**0.005**
Chest pain, n (%)	6 (10.5)	1 (1.8)	2.289	0.130
Nausea and vomiting, n (%)	5 (8.8)	5 (9.1)	0.00	1.00
Diarrhea, n (%)	2 (3.5)	3 (5.5)	0.002	0.967

Data are presented as means ± SD or number (%)

^a^The two groups (adults and children) have different age ranges, so a t-test is not appropriate to compare their means

^b^All % calculated for 57 or 55 patients, respectively, unless stated otherwise. In case % were calculated for less than the maximal number of patients, data for some patients were missing and the actual denominator is displayed

Abbreviations *NA* Not available

The main symptoms at admission were fever, cough, and sputum production in both groups. The children group had a significantly higher proportion and peak of fever (39.1 ± 0.7 °C vs 38.6 ± 0.7 °C, *p* = 0.004) than the adults group. However, the adult group had a higher proportion of expectoration, headache and sore throat (Table 1).

### Comparison of laboratory tests between the two groups

On admission, only a small number of patients had moderately elevated leukocyte and neutrophil counts. Decreased lymphocyte count was more common in adults (25% vs 1.9%, *p* = 0.001). Thrombocytosis was more common in children group (37.0% vs 10.7%, *p* = 0.001), and no thrombocytopenia was observed in both groups. C-reactive protein (CRP) and erythrocyte sedimentation rate (ESR) were increased in most patients, but procalcitonin (PCT) increased more in children (76.9% vs 13.5%, *p* < 0.001). Prothrombin time (PT) was slightly prolonged in a few adults, and a minor increase in D-dimer levels was observed in a small fraction of patients in both groups. Liver dysfunction was mild, with elevated liver enzymes and bilirubin in adults, elevated aspartate aminotransferase (AST) and normal bilirubin in children. Only 3 adults had mild elevated creatine, and there was no renal impairment in children. Adults had a higher incidence of hypoalbuminemia (31.6% vs 11.1%, *p* = 0.009), but the albumin levels were only marginally lower. Lactate dehydrogenase (LDH) was increased in most children (42, 77.8%) and a few adults (6, 11.3%) (*p* < 0.001**) **(Table 2).

**Table 2:** Laboratory results

Variables^a^	Parents group^b^ (*n* = 57)	Children group^b^ (*n* = 55)	χ^2^ value	*p* value
WBC (× 10^9^ per L; normal range for adults 3.5–9.5; for children 4–10.5)	6.9 ± 2.1	8.6 ± 3.3		
Increased, n (%)	7/56 (12.5)	10/54 (18.5)	0.762	0.383
Decreased, n (%)	0/56 (0)	2/54 (3.7)	0.547	0.459
Neu (× 10^9^ per L; normal range for adults 1.8–6.3; for children 1.08–5.8)	4.4 ± 1.8	4.9 ± 2.4		
Increased, n (%)	9/56 (16.1)	12/52 (23.1)	0.845	0.358
Lym (× 10^9^ per L; normal range for adults 1.1–3.2; for children 1.15–6)	1.7 ± 0.8	2.9 ± 1.6		
Increased, n (%)	2/56 (3.6)	6/52 (11.5)	1.469	0.226
Decreased, n (%)	14/56 (25)	1/52 (1.9)	12.006	**0.001**
Hb (g/L; normal range for adults 115–150; for children 110–149)	129 ± 14	124 ± 12		
Decreased, n (%)	6/56 (10.7)	7/54 (13.0)	0.133	0.715
PLT (× 10^9^ per L; normal range for adults 125–350; for children 100–378)	263 ± 64	310 ± 104		
Increased, n (%)	6/56 (10.7)	20/54 (37.0)	10.553	**0.001**
Decreased, n (%)	0/56 (0)	0/54 (0)		
CRP (mg/L; normal range < 3)	21.7 ± 27.6	15.1 ± 19.9		
Increased, n (%)	44/56 (78.6)	40 (72.7)	0.515	0.473
PCT (ng/mL; normal range 0–0.05)	0.05 ± 0.02	0.18 ± 0.32		
Increased, n (%)	7/52 (13.5)	40/52 (76.9)	42.275	** < .001**
ESR (mm/h; normal range 0–26)	40 ± 24	29 ± 18		
Increased, n (%)	33/45 (73.3)	7/11 (63.6)	0.071	0.790
PT (s; normal range 9–13)	12.2 ± 1.1	10.8 ± 0.8		
Increased, n (%)	11/55 (20)	0/16 (0)	2.413	0.120
D-dimer (mg/L; normal range for adults 0–0.55; for children 0–0.5)	0.42 ± 0.35	0.53 ± 0.35		
Increased, n (%)	9/56 (16.1)	6/17 (35.3)	1.892	0.169
ALT (U/L; normal range for adults 7–40; for children 9–50)	28 ± 35	18 ± 17		
Increased, n (%)	9/56 (16.1)	2 (3.6)	4.806	**0.028**
AST (U/L; normal range for adults 13–35; for children 15–40)	23 ± 16	32 ± 13		
Increased, n (%)	4/56 (7.1)	12 (21.8)	4.844	**0.028**
ALP (U/L; normal range for adults 35–100; for children 45–125)	68 ± 24	171 ± 53		
Increased, n (%)	4/56 (7.1)	21/54 (38.9)	15.776	** < .001**
ALB (g/L; normal range 40–55)	41.8 ± 3.4	43.1 ± 2.5		
Decreased, n (%)	18 (31.6)	6/54 (11.1)	6.855	**0.009**
TBIL (umol/L; normal range 0–23)	12.7 ± 15.9	5.6 ± 1.9		
Increased, n (%)	4 (7.0)	0/54 (0)	2.171	0.141
DBIL (umol/L; normal range 0–8)	5.2 ± 10.1	1.9 ± 0.7		
Increased, n (%)	3 (5.3)	0/54 (0)	1.262	0.261
BUN (mmol/L; normal range for adults 2.6–7.5; for children 3.1–8)	3.9 ± 1.2	3.6 ± 1.1		
Increased, n (%)	0 (0)	0/54 (0)	NA	NA
Cr (umol/L; normal range for adults 41–73; for children 57–97)	59 ± 14	31 ± 11		
Increased, n (%)	3 (5.3)	0 (0)	1.298	0.255
CK (U/L; normal range for adults 40–200; for children 50–310)	86 ± 56	108 ± 115		
Increased, n (%)	1/14 (7.1)	2/51 (3.9)	NA	0.523
LDH (U/L; normal range 120–250)	208 ± 37	319 ± 67		
Increased, n (%)	6/53 (11.3)	42/54 (77.8)	47.758	** < .001**

Data are presented as means ± SD or number (%)

^a^No t-test was performed to compare the two groups of children and adults, as the normal ranges of laboratory tests were different for the two groups

^b^All % calculated for 57 or 55 patients, respectively, unless stated otherwise. In case % were calculated for less than the maximal number of patients, data for some patients were missing and the actual denominator is displayed

*Abbreviations* *NA* Not available, *WBC* White blood cell, *Neu* Neutrophil, *Lym* Lymphocyte, *Hb* Hemoglobin, *PLT* Platelet, *CPR* C-reactive protein, *PCT* Procalcitonin, *ESR* Erythrocyte sedimentation rate, *PT* Prothrombin time, *ALT* Alanine aminotransferase, *AST* Aspartate aminotransferase, *ALP* Alkaline phosphatase, *ALB* Albumin, *TBIL* Total bilirubin, *DBIL* Direct bilirubin, *BUN* Blood urea nitrogen, *Cr* Creatinine, *CK* Creatine kinase, *LDH* Lactate dehydrogenase

### Comparison of imaging findings between the two groups

**Fig. 3:**
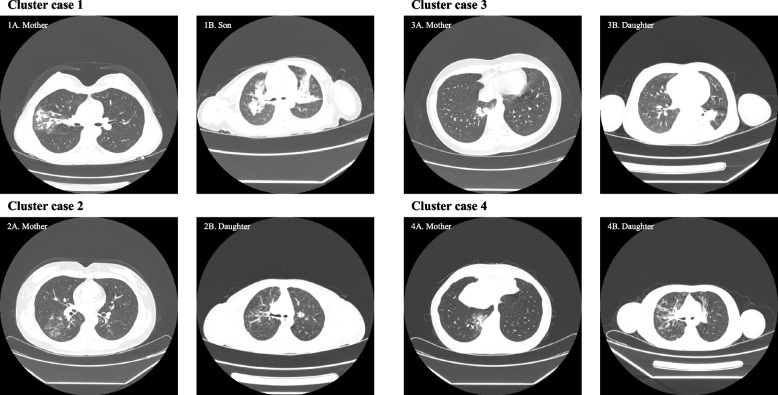
Chest CTs of four groups of clustered cases. Cluster case 1: CT of a 38-year-old mother and her 2-year-old son. The lung markings are clear and focal and patchy opacities are seen in the apical and posterior segments of the right upper lobe (1A). Patchy opacities are seen in the upper lobes of both lungs, with blurred margins (1B). Cluster case 2: CT of a 38-year-old mother and her 4-year-old daughter. There are increased bronchovascular shadows in the right lung, with bronchial wall thickening and peribronchial consolidation in the right lower lobe (2A). There are increased bronchovascular shadows in both lungs, with heterogeneous attenuation and multiple areas of bronchial wall thickening. Small nodular and patchy opacities are seen along the bronchovascular bundles (2B). Cluster case 3: CT of a 34-year-old mother and her 5-year-old daughter. Patchy and nodular high-density shadows are seen in the lower lobe of the right lung (3A). Increased bronchovascular shadows are seen in both lungs, with bronchial wall thickening, left lower lobe consolidation and patchy opacities (3B). Cluster case 4: CT of a 46-year-old mother and her 7-year-old daughter. Multiple nodular and patchy opacities are seen in the right lower lobe (4A). Increased bronchovascular shadows are seen in both lungs, with bronchial wall thickening and multiple patchy opacities around the bronchi, more prominent in the right lung (4B)

A total of 51 (89.5%) adults and 46 (88.5%) children were diagnosed with MPP. Patchy opacities on chest X-ray and computed tomography (CT) were the most frequent findings in both groups, affecting 47 (82.5%) adults and 44 (88%) children. Bronchial wall thickening was more prevalent in children (88% vs 40.4%, *p* < 0.001). Six adults and 11 children had consolidation and 3 had pleural effusion in both groups. Children tended to have bilateral pneumonia and adults tended to have unilateral pneumonia (Table 3). Figure 3 illustrates the typical CT findings of the four groups of family clusters of cases in this study.

**Table 3:** Chest X-ray and CT findings

Variables	Parents^a^ (*n* = 57)	Children^a^ (*n* = 55)	χ^2^ value	*p* value
Bronchial wall thickening, n (%)	23 (40.4)	44/50 (88)	25.835	** < .001**
Patchy opacities, n (%)	47 (82.5)	44/50 (88)	0.644	0.442
Ground-glass opacity, n (%)	9 (15.8)	3/50 (6)	2.564	0.109
Consolidation, n (%)	6 (10.5)	11/50 (22)	2.624	0.105
Unilateral infiltration, n (%)	34 (59.6)	15/52 (28.8)	10.427	**0.001**
Bilateral infiltration, n (%)	17 (29.8)	31/52 (59.6)	9.793	**0.002**
Pleural effusion, n (%)	3 (5.3)	3/52 (5.8)	0.00	1.00

Data are presented as number (%)

^a^All % calculated for 57 or 55 patients, respectively, unless stated otherwise. In case % were calculated for less than the maximal number of patients, data for some patients were missing and the actual denominator is displayed

*Abbreviations* *NA* Not available

### Pathogenic laboratory test results in both groups

MRMP was detected in 3 (60%) adults and 21 (52.5%) children. Co-infection was more prevalent in children than in adults (65.5% vs 22.8%, *p* < 0.001), with viral co-infection being the most frequent (41.8% in children). Other bacterial infections were detected in 3 (5.3%) adults and 20 (36.4%) children. Two adults and 3 children had fungal infection (Table 4).

**Table 4:** Pathogenic laboratory test results

Variables	Parents^a^ (*n* = 57)	Children^a^ (*n* = 55)	χ^2^ value	*p* value
MP-DNA, n (%)	14/17 (82.4)	39/53 (73.6)	0.167	0.683
MP-IgM, n (%)	43/53 (81.1)	42/51 (82.4)	0.026	0.872
mNGS, n (%)	12/13 (92.3)	13/13 (100)	NA	1.00
MRMP, n (%)	3/5 (60)	21/40 (52.5)	0.00	1.00
**Co-infection**	13 (22.8)	36 (65.5)	20.687	** < .001**
Viruses^b^, n (%)	10 (17.5)	23 (41.8)	7.936	**0.005**
Bacteria^c^, n (%)	3 (5.3)	20 (36.4)	16.591	** < .001**
Fungus^d^, n (%)	2 (3.5)	3 (5.5)	0.002	0.967

Data are presented as number (%)

^a^All % calculated for 57 or 55 patients, respectively, unless stated otherwise. In case % were calculated for less than the maximal number of patients, data for some patients were missing and the actual denominator is displayed

^b^Four adults tested positive for *SARS-CoV-2* and three for *parainfluenza virus*. Other viral infections included *respiratory syncytial virus*, *metapneumovirus*, *adenovirus*, and *rhinovirus*, each affecting one adult. *Parainfluenza virus* infected ten children, *rhinovirus* seven, *adenovirus* five, *Epstein-Barr virus* three, *influenza A virus* one, and *metapneumovirus* one

^c^One adult had *Haemophilus influenzae*, another had *Pseudomonas holleri*, and a third had *Legionella pneumophila*. *Haemophilus influenzae* affected fourteen children, *Streptococcus pneumoniae* seven, *Staphylococcus aureus* one, and *Klebsiella pneumoniae* one

^d^One adult had *Candida parapsilosis* and another had *Candida tropicalis*. One child had *Candida albicans*, another had *Candida parapsilosis*, and a third had *Aspergillus salviae*

Abbreviations *NA* Not available, *MP-DNA* *Mycoplasma pneumoniae* DNA detection, *MP-IgM* *Mycoplasma pneumoniae* IgM antibody detection, *mNGS* metagenomic next-generation sequencing detection, *MRMP* Macrolide-Resistant *Mycoplasma pneumoniae*

### Treatments and clinical outcomes

The antibiotic regimens varied between the two groups in some aspects. Macrolides were used in most children (96.4% vs 14.0%, *p* < 0.001) and quinolones were used in most adults (96.5% vs 3.6%, *p* < 0.001). Tetracyclines were used in 14 (25.5%) children. More children than adults switched from macrolides to alternative therapy (34.5% vs 7.0%, *p* < 0.001). Five children with MRMP infection changed from azithromycin to clindamycin, with good treatment effects. Besides antibiotic regimens, the two groups received different treatments. Glucocorticoids were given to 36 (65.5%) children and 5 (8.8%) adults, *p* < 0.001. Two children (3.6%) received immunoglobulin therapy, while none of the parents did.

Only one child with MRMP required intensive care unit (ICU) admission, where he received oxygen therapy, corticosteroids, intravenous immunoglobulin, meropenem and eventually had a good outcome. Children had a significantly longer fever duration than adults ((5.6 ± 2.2) days vs (4.1 ± 2.2) days, *p* = 0.002). None of the adults had SMPP, while 10 children (18.2%) fulfilled the diagnostic criteria for SMPP. No patients in this study required mechanical ventilation or died (Table 5).

**Table 5:** Treatments and clinical outcomes

**Variables**	**Parents** ^**b**^ ** (** *n* ** = 57)**	**Children** ^**b**^ ** (** ***n*** ** = 55)**	**χ** ^**2**^ ** /t value**	***p*** ** value**
Macrolides, n (%)	8 (14.0)	53 (96.4)	76.499	** < .001**
Tetracyclines, n (%)	1 (1.8)	14 (25.5)	13.555	** < .001**
Quinolones, n (%)	55 (96.5)	2 (3.6)	96.567	** < .001**
Other antibiotics^a^, n (%)	2 (3.5)	8 (14.5)	2.946	0.086
Methylprednisolone, n (%)	5 (8.8)	36 (65.5)	38.754	** < .001**
Intravenous immunogloblin, n (%)	0 (0)	2 (3.6)	0.546	0.460
Required oxygen, n (%)	13 (22.8)	15 (27.3)	0.298	0.585
ICU admission, n (%)	0 (0)	1 (1.8)	NA	0.491
Total duration of fever (d)	4.1 ± 2.2	5.6 ± 2.2	3.270	**0.002**
Length of stay in hospital (d)	8.0 ± 2.5	7.1 ± 2.4	-1.766	0.080
Change of macrolides to alternative therapy, n (%)	4 (7.0)	19 (34.5)	12.998	** < .001**
MPP, n (%)	51 (89.5)	46/52 (88.5)	0.028	0.866
SMPP, n (%)	0 (0)	10 (18.2)	9.254	**0.002**
Mechanical ventilation, n (%)	0 (0)	0 (0)	NA	NA
Death, n (%)	0 (0)	0 (0)	NA	NA

Data are presented as means ± SD or number (%)

^a^Other types of antibiotics are carbapenems (such as meropenem and imipenem) and aminoglycosides (such as etimicin)

^b^All % calculated for 57 or 55 patients, respectively, unless stated otherwise. In case % were calculated for less than the maximal number of patients, data for some patients were missing and the actual denominator is displayed

Abbreviations *NA* Not available, *ICU* Intensive care unit, *MPP* *Mycoplasma pneumoniae* pneumonia, *SMPP* Severe *Mycoplasma pneumoniae* pneumonia

## Discussion

This study investigated the clinical features of *Mycoplasma pneumoniae* (MP) infections in 63 families. We found that 65.5% of the adults required hospitalization, while almost all the children (94.2%) were hospitalized. Infections of MP and MRMP within familial clusters are primarily sourced from family case reports. The disease is highly contagious, spreading rapidly among all family members. This leads to a variety of individual responses that evolve with the progression of the disease, including conditions such as lymphoplasmacytic bronchiolitis and, in severe cases, death [9, 10]. Distinct variations in symptoms, laboratory findings, imaging results, treatment strategies, and outcomes have been observed between adults and children within familial clusters MP infection.

In our study, we observed that in 41 families, children were the first to be infected with MP. This could potentially be attributed to the higher frequency of exposure children might have in school or other community settings, which could lead to an earlier exposure to the pathogen. Additionally, adults generally have more robust immune systems than children, which might result in a longer incubation period before the onset of symptoms. On the other hand, in 15 families, adults were the first to contract MP. One possible explanation for this could be that children, especially younger ones, might not be able to articulate their symptoms as clearly as adults can. Consequently, their infections might not be recognized until some time after they have started showing symptoms.

The mean age of the children group was 6.3 ± 3.9 years, which is consistent with previous reports [11]. The caregivers had an average age of 35.1 ± 4.6 years, and most of them were female. A plausible explanation is the differences in immunity and social activities between men and women, and the fact that mother in Chinese families tend to care for children more and have closer contact with them [6, 12]. Both groups had similar clinical symptoms, with fever and cough being the most frequent on admission. We observed that children had higher and longer fever peaks than adults. Children with refractory MPP often have persistent fever. Monitoring the fever pattern in children is crucial for assessing the disease progression and outcome [13]. Previous studies showed that children with MP had a fever lasting 7–10 days [11, 14]. However, our patients with familial MP infection had a shorter fever duration than those reported in the literature, which was consistent with the lower proportion of lung consolidation in our cohort.

Our study showed that lymphopenia was more common in adults with MPP than in children. The hallmark of human MPP pathology was a prominent infiltration of lymphocytes in the peri-bronchovascular area, along with the presence of macrophages, neutrophils, and lymphocytes in the alveoli. Lymphocytes had a dual role in MPP, as they could either boost the host defense against MP or cause immune-mediated lung injury and complications [15]. Modulating the lymphocyte balance might be a potential strategy for MPP treatment. We noted that 37.0% children had thrombocytosis, a condition that previous studies have also reported and that was more prevalent in the convalescent phase, which might be associated with the inflammation stage or the age factor [16]. Most children had higher LDH levels than adults (*p* < 0.001). LDH is a cytoplasmic enzyme in all tissue cells that is released into the blood when cells are damaged or lysed. It can be used as a biomarker of tissue injury. LDH is a predictor of necrotizing pneumonia in children with mycoplasma pneumonia [14].

The radiological findings showed that the most common manifestation was patchy opacities, followed by bronchial wall thickening (more common in children). These findings are non-specific and similar to those reported in previous studies on MP [5, 12]. Several researchers have proposed that the bronchial wall thickening could serve as a diagnostic indicator [15]. The radiological patterns may reflect the pathogenesis of MPP, which involves attachment to the respiratory epithelium, induction of cytokines and chemokines, recruitment of inflammatory cells, and formation of exudates and necrosis [17].

Our study revealed a higher prevalence of co-infections in children compared to adults. This observation aligns with the laboratory findings and the severity of the disease, as a greater proportion of children exhibited elevated PCT levels. Specifically, 10 children (18.2%) were diagnosed with SMPP, and one of these cases required intensive care. The adults only had mild symptoms. According to previous research, co-infections, occurred in 8.2% of children with MP (mainly virus). This may increase the risk of SMPP [11, 18]. Prior research indicates that patients secondarily infected with COVID-19 within a family setting often exhibit milder symptoms [19]. It’s noteworthy that in our study, a significant majority of the adults (65.1%) were secondary infections. This could partially account for their less severe pneumonia compared to the children.

The therapeutic strategies varied between adults and children. Almost all children received macrolides, while most adults received quinolones. This may be due to the different guidelines and preferences for treating MPP in different age groups. The 2016 Chinese guidelines for the diagnosis and treatment of adult community-acquired pneumonia suggest quinolones or tetracyclines as the first-line antibiotics for adult MPP, and downgrade azithromycin to a second-line option [7]. Children were more prone to co-infections and may require other antibiotics. This study reported that 3 adults and 21 children had MRMP infections and most changed their antibiotics. Recent studies indicated that drug resistance did not affect the clinical features or the presence of pulmonary consolidation in MPP. MRMP infections only prolonged the fever duration and the hospital stay by 1–2 days [20]. Perhaps because of the immunomodulatory effects of macrolide antibiotics [15, 21], children who did not change their antibiotic regimen had favorable responses. Additionally, more children than adults received steroids (*p* < 0.001), which may be used to reduce inflammation or prevent complications such as bronchiolitis obliterans.

A major limitation of this study was the insufficient sample size to examine the risk factors of severe mycoplasma pneumonia in children and adults. In addition, this study is based on data from a single center and may not reflect the broader situation. Also, due to the infrequent testing of MRMP in adult patients, we were unable to provide comprehensive MRMP test results within the same family.

## Conclusion

This study discerned distinct clinical characteristics between adults and children within familial clusters MP infection. The differences underscore the variability in disease manifestation across age groups. While there existed a heightened risk of severe infection among children, the therapeutic outcomes for both adults and children were largely positive, attributable to the judicious application of antibiotics.

### Supplementary Information

**Additional file 1: Supplemental table 1. **Epidemic characteristics of MP infection in adults and children from Oct. 2022 to Oct. 2023. 

## Data Availability

No datasets were generated or analysed during the current study.

## Abbreviations

MP: *Mycoplasma pneumoniae*
MPP: *Mycoplasma pneumoniae* Pneumonia
MRMP: Macrolide-resistant *Mycoplasma pneumoniae*
SMPP: Severe *Mycoplasma pneumoniae* pneumonia
CRP: C-reactive protein
SAA: Serum amyloid A protein
ESR: Erythrocyte sedimentation rate
PCT: Procalcitonin
AST: Aspartate aminotransferase
ALT: Alanine transaminase
TBIL: Total bilirubin
DBIL: Direct bilirubin
ALP: Alkaline phosphatase
LDH: Lactate dehydrogenase
CT: Computed tomography
MSMP: Macrolide-susceptible *Mycoplasma pneumoniae*
ICU: Intensive care unit

## References

[1] Xue T. [Beware of children’s mycoplasma pneumonia in autumn and winter Experts advise timely identification and treatment]. Xin hua. Accessed 2023–09–03.

[2] Zhang L, Xiao Y, Zhang G (2023). Identification of priority pathogens for aetiological diagnosis in adults with community-acquired pneumonia in China: a multicentre prospective study. BMC Infect Dis.

[3] Atkinson TP, Balish MF, Waites KB (2008). Epidemiology, clinical manifestations, pathogenesis and laboratory detection of Mycoplasma pneumoniae infections. FEMS Microbiol Rev.

[4] Jacobs E, Ehrhardt I, Dumke R (2015). New insights in the outbreak pattern of Mycoplasma pneumoniae. Int J Med Microbiol.

[5] Saraya T, Watanabe T, Tsukahara Y (2017). The Correlation between Chest X-ray Scores and the Clinical Findings in Children and Adults with Mycoplasma pneumoniae Pneumonia. Intern Med.

[6] Lv YT, Sun XJ, Chen Y, Ruan T, Xu GP, Huang JA (2022). Epidemic characteristics of Mycoplasma pneumoniae infection: a retrospective analysis of a single center in Suzhou from 2014 to 2020. Ann Transl Med.

[7] Branch CMARD (2016). Chinese guidelines for diagnosis and treatment of adult community-acquired pneumonia (2016 edition). Chin J Tuberc Respir Dis.

[8] National Health Commission of the People’s Republic of China. [Guidelines for the diagnosis and treatment of Mycoplasma pneumoniae pneumonia in children(2023 edition)]. Int J Epidemiol Infect Dis. 2023;50(2):79–85.

[9] Kannan TR, Hardy RD, Coalson JJ (2012). Fatal outcomes in family transmission of Mycoplasma pneumoniae. Clin Infect Dis.

[10] Tsai V, Pritzker BB, Diaz MH (2013). Cluster of macrolide-resistant Mycoplasma pneumoniae infections in Illinois in 2012. J Clin Microbiol.

[11] Wang L, Xie Q, Xu S (2023). The role of flexible bronchoscopy in children with Mycoplasma pneumoniae pneumonia. Pediatr Res.

[12] Ren Y, Wang Y, Liang R (2022). Development and validation of a nomogram for predicting Mycoplasma pneumoniae pneumonia in adults. Sci Rep.

[13] Jang MS, Kim BG, Kim J (2021). Prediction model for prolonged fever in patients with Mycoplasma pneumoniae pneumonia: a retrospective study of 716 pediatric patients. BMC Pulm Med.

[14] Luo Y, Wang Y (2023). Risk Prediction Model for Necrotizing Pneumonia in Children with Mycoplasma pneumoniae Pneumonia. J Inflamm Res.

[15] Saraya T, Kurai D, Nakagaki K (2014). Novel aspects on the pathogenesis of Mycoplasma pneumoniae pneumonia and therapeutic implications. Front Microbiol.

[16] Youn YS, Lee KY, Hwang JY (2010). Difference of clinical features in childhood Mycoplasma pneumoniae pneumonia. BMC Pediatr.

[17] Parrott GL, Kinjo T, Fujita J (2016). A Compendium for Mycoplasma pneumoniae. Front Microbiol.

[18] Li F, Zhang Y, Shi P (2022). Mycoplasma pneumoniae and Adenovirus Coinfection Cause Pediatric Severe Community-Acquired Pneumonia. Microbiol Spectr.

[19] Diao KY, Zhang XC, Huang S (2021). Features of family clusters of COVID-19 patients: A retrospective study. Travel Med Infect Dis.

[20] Yen MH, Yan DC, Wang CJ (2023). The clinical significance of and the factors associated with macrolide resistance and poor macrolide response in pediatric Mycoplasma pneumoniae infection: A retrospective study. J Microbiol Immunol Infect.

[21] Lee H, Yun KW, Lee HJ, Choi EH (2018). Antimicrobial therapy of macrolide-resistant Mycoplasma pneumoniae pneumonia in children. Expert Rev Anti Infect Ther.

