# Supplementary material for: Comparison of the clinical characteristics in parents and their children in a series of family clustered Mycoplasma pneumoniae infections
Source: BMC Pulm Med. 2024 Mar 4;24:107. doi: 10.1186/s12890-024-02922-0 (PMC10910824; doi:10.1186/s12890-024-02922-0)
Supplement: Supplementary file 1 — Additional file 1: Supplemental table 1. Epidemic characteristics of MP infection in adults and children from Oct. 2022 to Oct. 2023. [file 12890_2024_2922_MOESM1_ESM.docx]

**Supplemental table 1.** Epidemic characteristics of MP infection in adults and children from Oct. 2022 to Oct. 2023.

| **Variables** | **Adults (n/N, %)** | **Children (n/N, %)** |
| --- | --- | --- |
| Month |  |  |
| Oct. 2022 | 22/258 (8.5) | 212/761 (27.9) |
| Nov. 2022 | 15/242 (6.2) | 119/480 (24.8) |
| Dec. 2022 | 87/781 (11.1) | 135/738 (18.3) |
| Jan. 2023 | 74/621 (11.9) | 106/351 (30.2) |
| Feb. 2023 | 21/360 (5.8) | 255/725 (35.2) |
| Mar. 2023 | 22/384 (5.7) | 592/1741 (34.0) |
| Apl. 2023 | 61/439 (13.9) | 562/1602 (35.1) |
| May 2023 | 56/556 (10.1) | 436/1625 (26.8) |
| Jun. 2023 | 74/459 (16.1) | 657/1752 (37.5) |
| Jul. 2023 | 54/416 (13.0) | 973/1762 (55.2) |
| Aug. 2023 | 81/557 (14.5) | 1005/1630 (61.7) |
| Sep. 2023 | 105/578 (18.2) | 981/1740 (56.4) |
| Oct. 2023 | 79/807 (9.8) | 1404/2031 (69.1) |

Abbreviations: n, the number of patients with positive laboratory tests for Mycoplasma pneumoniae; N, total number of patients with laboratory tests for Mycoplasma pneumoniae
